# Weaker Effects of Educational Attainment on Chronic Medical Conditions in American Indian Alaska Native, Black, and Latino Adults: National Health Interview Survey 2023

**DOI:** 10.31586/ojms.2025.1150

**Published:** 2025-01-23

**Authors:** Shervin Assari, Hossein Zare

**Affiliations:** 1 Department of Internal Medicine, Charles R Drew University of Medicine and Science, Los Angeles, CA, USA; 2 Department of Urban Public Health, Charles R Drew University of Medicine and Science, Los Angeles, CA, USA; 3 Marginalized-Related Diminished Returns (MDRs) Research Center, Los Angeles, CA, USA; 4 Department of Health Policy and Management, Johns Hopkins Bloomberg School of Public Health, Baltimore, MD, USA; 5 School of Business, University of Maryland Global Campus (UMGC), Adelphi, MD, USA

**Keywords:** Chronic Medical Conditions, Health Disparities, Socioeconomic Status, Minorities’ Diminished Returns (MDRs), Racial and Ethnic Inequities, Educational Attainment, Structural Racism

## Abstract

**Background:**

Chronic medical conditions are major drivers of healthcare spending, morbidity, and mortality in the United States, as well as critical indicators of health disparities. The disproportionately high rates of chronic medical conditions among Black, Latino, and American Indian and Alaska Native adults compared to non-Latino Whites highlight the urgent need to examine the factors contributing to these disparities. While higher socioeconomic status is generally associated with better health outcomes, this benefit may be diminished for racialized and minoritized populations.

**Objective:**

This study investigates the protective effects of educational attainment and income-to-poverty ratio on the prevalence of chronic medical conditions and examines whether these effects vary across racial and ethnic groups, specifically among Black, Latino, and American Indian and Alaska Native adults compared to non-Latino White adults.

**Methods:**

Using data from the 2023 National Health Interview Survey (NHIS), this cross-sectional study analyzed the association between educational attainment and chronic medical conditions across racial and ethnic groups. Logistic regression models were employed to assess whether the strength of the relationship between education and chronic medical conditions differed by racial/ethnic group, controlling for key demographic and socioeconomic covariates. Sample size was 29,373 which was reflective of 256,566,689 US population.

**Results:**

Consistent with the theory of Minorities’ Diminished Returns, findings showed that the protective effects of higher educational attainment on chronic medical conditions were significantly weaker for Black, Latino, and American Indian and Alaska Native adults than for their non-Latino White counterparts. Even among individuals with higher education, Black, Latino, and American Indian and Alaska Native adults faced elevated risks of chronic medical conditions.

**Conclusion:**

While educational attainment generally reduces the prevalence of chronic medical conditions, this protective effect is moderated by racial and ethnic background. Structural barriers limit the health benefits of educational attainment. This underscores the need for policies that address structural inequities—such as low- quality education and occupational segregation—that constrain the protective health effects of educational attainment for minoritized groups.

## 1. Introduction

Chronic medical conditions are the leading contributors to healthcare spending [1–3]. They are also major causes of morbidity and mortality in the United States and worldwide, constituting a significant public health burden [4–7]. These conditions require extensive, often costly, long-term management and consume most of the healthcare budgets in developed, high-income, and industrial countries [8,9].

Chronic medical conditions are not distributed at random [10,11]; they disproportionately impact racialized populations. Historically minoritized groups, particularly Black, Latino, and American Indian and Alaska Native adults, report higher prevalence rates of chronic medical conditions than their non-Latino White counterparts [12–15]. As such, chronic medical conditions represent a key indicator of health disparities, reflecting not only individual risk factors but also deep-rooted structural inequities that shape health outcomes across the life course [16].

Education and income-to-poverty ratio are widely acknowledged as essential determinants of health, with extensive literature demonstrating that individuals with higher socioeconomic status—often indicated by educational attainment and income— experience lower prevalence of chronic medical conditions. Scholars such as Braveman [17–23], Marmot [24–28], Link [29–33], Ross [34–36], Mirowsky [34,35,37,38], and Williams [39–44] have extensively theorized and documented the critical role of socioeconomic status in promoting population health. Education can improve health outcomes through various pathways, such as increased access to preventive care, greater health literacy, and the ability to engage in health-promoting behaviors like regular exercise, balanced nutrition, and effective self-care [34–36]. Individuals with higher socioeconomic status are also more likely to occupy healthier, less physically taxing jobs, live in neighborhoods with better food access, and secure health insurance coverage, all of which contribute to reduced risks of developing chronic medical conditions [34,35,37,38].

However, the benefits of socioeconomic status are not universally distributed across racial and ethnic groups [45,46]. Theories of health inequality, including Minorities’ Diminished Returns [47], posit that the health benefits of education and income-to- poverty ratio are less pronounced among Black, Latino, and American Indian and Alaska Native populations. Structural racism, social stratification, and residential and occupational segregation limit the protective effects of socioeconomic status, with minoritized individuals frequently facing inequitable access to quality education, employment, and healthcare. Consequently, the expected health benefits of educational attainment may be weaker among Black, Latino, and American Indian and Alaska Native adults, contributing to persistent disparities in chronic medical conditions [47–56].

This study aims to (1) examine the association between educational attainment and chronic medical conditions across racial and ethnic groups, and (2) test the hypothesis that the protective effects of education on chronic medical conditions prevalence are diminished among Black, Latino, and American Indian and Alaska Native adults relative to non-Latino Whites. Using the latest data from the 2023 National Health Interview Survey (NHIS) [57], we assess whether disparities in chronic medical conditions persist despite higher levels of education among minoritized populations, thereby highlighting the need for policy interventions that address structural barriers to equitable health outcomes.

## 2. Methods

### 2.1. National Health Interview Survey (NHIS)

The National Health Interview Survey (NHIS) is the principal source of health data for the civilian, noninstitutionalized U.S. population, conducted by the National Center for Health Statistics (NCHS) since 1957. Authorized by the National Health Survey Act of 1956, NHIS continuously gathers data on health and disability across various demographics and socioeconomic groups, supporting research and policy initiatives within the Department of Health and Human Services and beyond [57].

### 2.2. NHIS 2023

In 2023, the NHIS conducted 29,522 Sample Adult interviews and 7,692 Sample Child interviews, achieving response rates of 47.0% and 44.9%, respectively. Data were collected through both in-person and telephone interviews, with 54.5% of interviews conducted at least partially by phone, similar to 2022 but higher than pre-pandemic levels in 2019 [58].

### 2.3. Design

NHIS is a cross-sectional, continuous survey covering the U.S. civilian noninstitutionalized population in the 50 states and the District of Columbia. Data are collected throughout the year, allowing monthly samples to be nationally representative. To manage costs and logistics, NHIS employs a geographically clustered sampling design [58].

### 2.4. Sampling

The sampling process begins by partitioning the United States into 1,689 geographic areas, based on counties or groups of counties. These areas are further stratified by population density in some states, while smaller states and the District of Columbia remain unstratified. Clusters within each stratum are selected proportionally, ensuring a representative national sample [58].

### 2.5. Sample

The NHIS targets individuals in households and noninstitutional group quarters, such as homeless shelters and group homes, but excludes active-duty military personnel, persons in long-term care institutions, and residents in correctional facilities [58].

### 2.6. Process and Interview

The U.S. Census Bureau conducts NHIS interviews under contract, deploying approximately 864 trained interviewers nationwide. Interviewers use computer-assisted personal interviewing (CAPI) technology, which facilitates question routing, real-time data entry, and data validation. Respondents receive an advance letter explaining NHIS participation, ensuring informed, voluntary consent. Interviews are conducted primarily in person, with follow-ups or special requests handled by phone [58].

### 2.7. Analytical Sample for this Paper

This paper used a sample of 29,373 participants, representative of 256,566,689. Eligibility criteria included being an adult (age >=18) from any race/ethnicity.

### 2.8. Measures

#### Independent Variables: Educational Attainment:

Participants reported their highest level of education as one of the following categories: 00 (Never attended/kindergarten only), 01 (Grade 1-11), 02 (12th grade, no diploma), 03 (GED or equivalent), 04 (High school graduate), 05 (Some college, no degree), 06 (Associate degree: occupational, technical, or vocational program), 07 (Associate degree: academic program), 08 (Bachelor’s degree, e.g., BA, BS, BBA), 09 (Master’s degree, e.g., MA, MS, MBA), and 10 (Professional or doctoral degree, e.g., MD, JD, PhD). For analysis, we consolidated these categories into three groups: (1) Less than high school diploma (categories 0-3), (2) Some college (categories 4-7), and (3) College degree or higher (categories 8+). Educational attainment was treated as a three-level categorical variable, with “less than high school diploma” as the reference category, and “some college” and “college degree or higher” as the other two levels compared to this reference.

#### Income-to-Poverty Ratio:

We used the 0-11 continuous measure of income-to- poverty ratio from the NHIS data and recoded it into a three-level variable representing low [reference group], middle, and high income-to-poverty ratios.

#### Outcome: Chronic Medical Conditions:

We defined chronic medical conditions as a dichotomous (binary) outcome, coded as 0 for no chronic medical conditions and 1 for the presence of any chronic medical condition, regardless of type. The conditions included were hypertension, high cholesterol, coronary artery disease, heart attack, stroke, asthma, cancer, epilepsy, chronic obstructive pulmonary disease (COPD), diabetes, Alzheimer’s disease, hepatitis, and arthritis. These conditions were self-reported, based on participants’ responses to whether a doctor had ever informed them that they had such a condition.

#### Covariates:

Gender (female=0, male =1), age (years), and marital status (other =0, married =1). All covariates were self-report.

#### Moderator: Race/Ethnicity:

Participants self-identified their race/ethnicity as Non- Latino White, Black, Hispanic, American Indian and Alaska Native (AIAN), or Other/Mixed (which included individuals identifying as Asian). This variable was treated as a nominal variable, with Non-Latino White as the reference group.

### 2.9. Ethics

This study was conducted in compliance with ethical standards, ensuring the protection and confidentiality of participant information. All data were collected anonymously, and no identifying information was retained. The study adhered to the ethical principles outlined in the Declaration of Helsinki, emphasizing respect, beneficence, and justice in research practices. Written informed consent was obtained from all participants prior to their involvement in the study, following a clear explanation of the study’s purpose, procedures, potential risks, and benefits. Institutional Review Board (IRB) approval was obtained for NHIS to ensure that all ethical guidelines were rigorously followed throughout the research process. Current analysis used fully deidentified existing data and did not need a full IRB review.

### 2.10. Statistical Analysis

We conducted all analyses using Stata, accounting for survey design variables, including survey weights and strata, to ensure accurate representation of the U.S. adult population. Initially, we ran a logistic regression model without interaction terms to test the additive effects of race/ethnicity, educational attainment, and income-to-poverty ratio on chronic medical conditions, adjusting for age, gender, and marital status as covariates. We then conducted a second logistic regression model that included interaction terms between race/ethnicity and both educational attainment and income-to-poverty ratio. These models retained all covariates from the initial model. For each logistic regression model, we reported the odds ratios (OR), standard errors (SE), 95% confidence intervals (CI), and p-values. All results presented are representative of Non-Latino White and American Indian and Alaska Native adults.

## 3. Results

Table 1 presents the descriptive statistics for the sample population. The mean age of participants was 48.189 years (SE = 0.158). In terms of race/ethnicity, 61.974% (SE = 0.745) of the sample identified as Non-Latino White, 11.805% (SE = 0.435) as Non-Latino Black, 17.305% (SE = 0.633) as Latino White, 1.27% (SE = 0.16) as American Indian or Alaska Native (AIAN), and 7.646% (SE = 0.322) as Other/Mixed.

For gender, 51.207% (SE = 0.354) of participants were women, while 48.792% (SE = 0.354) were men. Marital status data indicate that 50.787% (SE = 0.412) of participants were not married, and 49.213% (SE = 0.412) were married. Regarding educational attainment, 12.814% (SE = 0.356) had less than a high school education, 54.112% (SE = 0.419) were high school graduates, and 33.074% (SE = 0.474) were college graduates.

Income-to-poverty ratio data reveal that 32.232% (SE = 0.521) of participants were in the low-income group, 42.23% (SE = 0.381) in the mid-income group, and 25.538% (SE = 0.46) in the high-income group. Concerning chronic medical conditions, 39.382% (SE = 0.393) reported no chronic conditions, while 60.618% (SE = 0.393) reported having at least one chronic condition.

Table 2 shows the summary of the logistic regression (Model 1) with chronic medical conditions (any) as the outcome. Educational attainment showed protective effects. College graduates had lower odds of chronic medical conditions compared to those with less than a high school diploma (OR = 0.84, 95% CI: 0.74 - 0.97, p = 0.014), while having only a high school diploma did not significantly reduce the odds (OR = 1.02, 95% CI: 0.90 - 1.15, p = 0.767).

Income-to-poverty ratio also played a protective role. Individuals with mid-level income had lower odds of chronic medical conditions compared to those in the low- income group (OR = 0.79, 95% CI: 0.72 - 0.86, p < 0.001), and individuals with high income had even lower odds (OR = 0.74, 95% CI: 0.67 - 0.82, p < 0.001). The intercept was statistically significant (OR = 0.13, 95% CI: 0.11 - 0.16, p < 0.001), indicating a low baseline probability of chronic medical conditions in the reference category.

Compared to non-Hispanic Whites, Hispanic adults had significantly lower odds of having chronic medical conditions (OR = 0.66, 95% CI: 0.59 - 0.72, p < 0.001), as did individuals in the “Other” racial category (OR = 0.66, 95% CI: 0.57 - 0.75, p < 0.001). However, there was no significant difference between Black adults and non-Hispanic Whites (OR = 0.98, 95% CI: 0.88 - 1.09, p = 0.656), nor between AIAN adults and non- Hispanic Whites (OR = 1.10, 95% CI: 0.80 - 1.53, p = 0.550).

Age was positively associated with the presence of chronic medical conditions, with each additional year associated with a slight increase in the odds of having a chronic condition (OR = 1.06, 95% CI: 1.06 - 1.07, p < 0.001). Gender did not show a significant association, as males had similar odds of chronic medical conditions compared to females (OR = 1.04, 95% CI: 0.97 - 1.11, p = 0.255). Additionally, marital status was not significantly associated with chronic conditions, with married individuals having comparable odds to unmarried individuals (OR = 0.94, 95% CI: 0.88 - 1.01, p = 0.088).

Table 3 shows summary of a logistic regression analysis with chronic medical conditions as the outcome (Model 2). Educational attainment had a protective effect, with high school diploma holders having lower odds of CMCs compared to those with less than a high school diploma (OR = 0.79, 95% CI: 0.66 - 0.95, p = 0.013). College graduates showed even lower odds (OR = 0.63, 95% CI: 0.52 - 0.76, p < 0.001). Income-to-poverty ratio further reduced the odds of CMCs, with individuals in the mid-income category having lower odds compared to the low-income group (OR = 0.72, 95% CI: 0.64 - 0.81, p < 0.001), and high-income individuals having even lower odds (OR = 0.67, 95% CI: 0.59 - 0.75, p < 0.001).

The interaction terms revealed that the protective effects of educational attainment varied by race/ethnicity. For Black adults with a college education, the odds of CMCs were higher than their non-Hispanic White counterparts (OR = 1.72, 95% CI: 1.16 - 2.55, p = 0.007). Similarly, Hispanic college graduates had higher odds of CMCs than White college graduates (OR = 1.79, 95% CI: 1.29 - 2.48, p = 0.001), as did AIAN college graduates (OR = 4.45, 95% CI: 1.67 - 11.89, p = 0.003). For those with only a high school diploma, the odds were also elevated for Hispanic adults (OR = 1.47, 95% CI: 1.10 - 1.95, p = 0.008) and for AIAN adults (OR = 3.42, 95% CI: 1.62 - 7.25, p = 0.001), relative to their non-Hispanic White counterparts. In terms of the interaction between race/ethnicity and income-to-poverty ratio, individuals in the “Other” racial category with high income showed higher odds of CMCs compared to their low-income non-Hispanic White counterparts (OR = 1.55, 95% CI: 1.09 - 2.21, p = 0.015). Other interaction terms between race/ethnicity and income levels did not reach statistical significance. The intercept was statistically significant (OR = 0.18, 95% CI: 0.14 - 0.22, p < 0.001), indicating a low baseline probability of chronic medical conditions in the reference category.

In addition, age was positively associated with CMCs, with each additional year increasing the odds (OR = 1.06, 95% CI: 1.06 - 1.07, p < 0.001). Gender was not significantly associated with CMCs, as males had similar odds to females (OR = 1.04, 95% CI: 0.97 - 1.11, p = 0.252). Marital status was also not significantly associated with CMCs (OR = 0.95, 95% CI: 0.89 - 1.02, p = 0.165).

## 4. Discussion

Using data from the 2023 National Health Interview Survey (NHIS), this study aimed to examine the association between educational attainment and the prevalence of chronic medical conditions overall, and also by race and ethnicity. We first hypothesized a protective effect of higher education on chronic medical conditions overall. Then we expected this protective effect to be weaker among Black, Latino, and American Indian and Alaska Native adults compared to non-Latino Whites. According to the Minorities’ Diminished Returns framework, structural barriers limit the full health benefits typically associated with educational attainment. If our hypothesis confirmed, racial and ethnic disparities in the prevalence of chronic medical conditions persist despite even when levels of education are high. This will highlight the need to address structural inequalities that undermine the health benefits of education for these groups.

Our first finding demonstrated an inverse association between educational attainment and chronic medical conditions. This relationship may be explained by the wide range of benefits associated with higher education, including reduced stress, improved living conditions, better employment opportunities, higher income, enhanced access to healthcare services, and healthier behaviors such as better nutrition, regular exercise, and lower rates of tobacco and drug use. These are explained by Braveman [17– 23], Marmot [24–28], Link [29–33], Ross [34–36], Mirowsky [34,35,37,38], and Williams [39–44].

The findings from this study contribute to a growing body of evidence supporting the Minorities’ Diminished Returns framework [47–56,59–65], which posits that the health benefits of socioeconomic resources are unevenly distributed across racial and ethnic groups. Consistent with our hypotheses, the protective effects of higher educational attainment on chronic medical conditions were significantly weaker among Black, Latino, and American Indian and Alaska Native adults compared to non-Latino Whites. These findings underscore the limitations of education as a protective factor against chronic medical conditions in the presence of pervasive structural barriers affecting marginalized populations.

One potential explanation for these diminished returns lies in the differential quality of educational resources available to Black, Latino, and American Indian and Alaska Native communities. Research indicates that schools in minoritized communities often lack resources, experienced teachers, and access to advanced coursework, which may limit the protective health benefits traditionally associated with higher education. Furthermore, structural racism in the labor market frequently channels Black, Latino, and American Indian and Alaska Native individuals into low-wage, high-stress jobs with fewer health benefits and limited upward mobility, diminishing the health-promoting potential of their educational achievements.

Additionally, residential segregation often restricts access to neighborhoods with high-quality healthcare services, nutritious food options, and safe recreational spaces, further eroding the health advantages of education among minoritized populations. This spatial inequality perpetuates a cycle in which the benefits of socioeconomic resources, including education, are compromised by environmental and social constraints.

### 4.1. Clinical Implications

The findings from this study highlight critical considerations for clinicians in addressing chronic medical conditions among high- socioeconomic status individuals from minoritized backgrounds. Clinicians should not assume that high socioeconomic status universally translates to lower health needs, as the protective effects of socioeconomic status on health are often attenuated among Black, Latino, and American Indian and Alaska Native patients. Relying solely on socioeconomic status as an indicator of health risk may lead to missed or delayed detection of chronic medical conditions in these groups, as socioeconomic status does not fully capture the social and structural adversities that persist despite higher education or income levels. Instead, clinicians should consider socioeconomic status as a nuanced, context-dependent factor, going beyond simple checklists to assess patients’ unique challenges, such as occupational stress, neighborhood context, and access to quality healthcare and preventive resources. Incorporating these broader, intersectional factors into clinical assessments could improve early detection and targeted management of chronic medical conditions, particularly in minoritized populations whose health outcomes may not align with typical socioeconomic status-based expectations.

### 4.2. Policy Implications

Our findings highlight the need for policy interventions that go beyond individual- level factors like education to address the structural inequities constraining the health benefits of socioeconomic status for Black, Latino, and American Indian and Alaska Native adults. Policies aimed at improving educational quality in minoritized communities, reducing occupational segregation, and expanding access to affordable healthcare and safe neighborhoods could mitigate the effects of MDRs on health disparities. Additionally, targeted efforts to improve health literacy and access to preventive services in underserved communities may help to counterbalance the weaker protective effects of education on chronic medical conditions.

### 4.3. Limitations

This study is not without limitations. The cross-sectional nature of NHIS data [58,66– 72] precludes the ability to draw causal inferences, and self-reported measures of chronic medical conditions may introduce reporting biases. Future research should explore these relationships longitudinally to better understand how MDRs evolve over time and across generations.

### 4.4. Future Research Directions

Future research should explore several avenues to deepen our understanding of the diminished returns of education on chronic medical conditions among minoritized populations. First, longitudinal studies are needed to track these disparities over time and across different life stages to better capture the cumulative impact of educational attainment on health outcomes. This approach could reveal whether and how the protective effects of education on chronic medical conditions shift with age and other social transitions, particularly for Black, Latino, and American Indian and Alaska Native individuals. Additionally, qualitative research could provide valuable insights into how individuals from these groups perceive and experience the barriers that weaken the health benefits of their educational achievements. Another important area for future research is the exploration of intersectional factors, such as gender, immigrant status, and geographic location, which may further modify the relationship between education and health. Finally, comparative studies across countries with different social welfare and educational systems could help identify which policies or programs are most effective in minimizing disparities in the health returns of education, offering evidence-based guidance for policymakers in the United States.

## 5. Conclusion

In summary, while educational attainment is associated with reduced prevalence of chronic medical conditions, this protective effect is significantly weaker for Black, Latino, and American Indian and Alaska Native adults than for non-Latino Whites. These findings underscore the limitations of education as a stand-alone solution to health disparities and point to the urgent need for structural interventions that address the social and environmental barriers limiting health equity across racial and ethnic groups.

## Figures and Tables

**Table 1.** Descriptive Data Overall

	Mean	SE
Age	48.189	0.158
		
	%	SE
Race/Ethnicity		
Non-Latino White	61.974	0.745
Non-Latino Black	11.805	0.435
Latino White	17.305	0.633
AIAN	1.27	0.16
Other/Mixed	7.646	0.322
Gender		
Women	51.207	0.354
Men	48.792	0.354
Marital Status		
Not Married	50.787	0.412
Married	49.213	0.412
Educational Attainment		
Less than High School Graduate	12.814	0.356
High School Graduate	54.112	0.419
College Graduate	33.074	0.474
Income to Poverty Ratio		
Low	32.232	0.521
Mid	42.23	0.381
High	25.538	0.46
Chronic Medical Conditions		
No	39.382	0.393
Yes	60.618	0.393

**Table 2.** Summary of Logistic Regression (Model 1)

	OR	SE	95%	CI	p
Race/Ethnicity					
Non-Hispanic White	Ref.				
Black	0.98	0.05	0.88	1.09	0.656
Hispanic	0.66	0.03	0.59	0.72	< 0.001
AIAN	1.10	0.18	0.80	1.53	0.550
Other	0.66	0.05	0.57	0.75	< 0.001
age	1.06	0.00	1.06	1.07	< 0.001
male	1.04	0.04	0.97	1.11	0.255
married	0.94	0.03	0.88	1.01	0.088
Educational Attainment					
Less than High School Diploma	Ref.				
High School Diploma	1.02	0.06	0.90	1.15	0.767
College Graduate	0.84	0.06	0.74	0.97	0.014
Income to Poverty Ratio					
Low	Ref.				
Mid	0.79	0.04	0.72	0.86	< 0.001
High	0.74	0.04	0.67	0.82	< 0.001
Intercept	0.13	0.01	0.11	0.16	< 0.001

Note: OR = Odds Ratio; SE = Standard Error; CI = Confidence Interval; Ref. = Reference category

**Table 3.** Summary of Logistic Regression (Model 2)

	OR	SE	95%	CI	p
Race/Ethnicity					
Non-Hispanic White	Ref.				
Black	0.61	0.11	0.44	0.86	0.005
Hispanic	0.40	0.05	0.31	0.51	< 0.001
AIAN	0.31	0.10	0.16	0.60	0.001
Other	0.35	0.10	0.20	0.62	< 0.001
age	1.06	0.00	1.06	1.07	< 0.001
male	1.04	0.04	0.97	1.11	0.252
married	0.95	0.03	0.89	1.02	0.165
Educational Attainment					
Less than High School Diploma	Ref.				
High School Diploma	0.79	0.07	0.66	0.95	0.013
College Graduate	0.63	0.06	0.52	0.76	< 0.001
Income to Poverty Ratio					
Low	Ref.				
Mid	0.72	0.04	0.64	0.81	< 0.001
High	0.67	0.04	0.59	0.75	< 0.001
Race/Ethnicity x Educational Attainment					
Black x High School Diploma	1.39	0.25	0.98	1.99	0.068
Black x College Graduate	1.72	0.34	1.16	2.55	0.007
Hispanic x High School Diploma	1.47	0.21	1.10	1.95	0.008
Hispanic x College Graduate	1.79	0.30	1.29	2.48	0.001
AIAN x High School Diploma	3.42	1.31	1.62	7.25	0.001
AIAN x College Graduate	4.45	2.23	1.67	11.89	0.003
Other x High School Diploma	1.59	0.48	0.88	2.86	0.126
Other x College Graduate	1.52	0.44	0.85	2.70	0.154
Race/Ethnicity x Income to Poverty Ratio					
Black x Mid	1.16	0.16	0.89	1.51	0.270
Black x High	1.15	0.19	0.84	1.59	0.382
Hispanic x Mid	1.23	0.14	0.99	1.54	0.067
Hispanic x High	1.19	0.17	0.90	1.59	0.227
AIAN x Mid	1.38	0.44	0.74	2.57	0.310
AIAN x High	0.91	0.40	0.38	2.18	0.833
Other x Mid	1.15	0.20	0.81	1.63	0.438
Other x High	1.55	0.28	1.09	2.21	0.015
Intercept	0.18	0.02	0.14	0.22	< 0.001

Note: OR = Odds Ratio; SE = Standard Error; CI = Confidence Interval; Ref. = Reference category
